# Polyphyllin I alleviates neuroinflammation after cerebral ischemia–reperfusion injury via facilitating autophagy-mediated M2 microglial polarization

**DOI:** 10.1186/s10020-024-00828-5

**Published:** 2024-05-14

**Authors:** Chunyang Kang, Qiuling Sang, Dingxi Liu, Libo Wang, Jia Li, Xiaoyang Liu

**Affiliations:** 1 Department of Neurology, China-Japan Union Hospital of Jilin University, No. 126 Xiantai Street, Changchun, 130000 China; 2 Department of Neuroelectrophysiology, China-Japan Union Hospital of Jilin University, Changchun, 130000 China; 3 Department of Clinical Medicine, Zunyi Medical University, Zhuhai, 519041 China

**Keywords:** Cerebral ischemia, Microglial polarization, Polyphyllin I (PPI), Autophagy, NLRP3 inflammasome

## Abstract

Microglial activation and polarization play a central role in poststroke inflammation and neuronal damage. Modulating microglial polarization from pro-inflammatory to anti-inflammatory phenotype is a promising therapeutic strategy for the treatment of cerebral ischemia. Polyphyllin I (PPI), a steroidal saponin, shows multiple bioactivities in various diseases, but the potential function of PPI in cerebral ischemia is not elucidated yet. In our study, the influence of PPI on cerebral ischemia–reperfusion injury was evaluated. Mouse middle cerebral artery occlusion (MCAO) model and oxygen–glucose deprivation and reoxygenation (OGD/R) model were constructed to mimic cerebral ischemia–reperfusion injury in vivo and in vitro. TTC staining, TUNEL staining, RT-qPCR, ELISA, flow cytometry, western blot, immunofluorescence, hanging wire test, rotarod test and foot-fault test, open-field test and Morris water maze test were performed in our study. We found that PPI alleviated cerebral ischemia–reperfusion injury and neuroinflammation, and improved functional recovery of mice after MCAO. PPI modulated microglial polarization towards anti-inflammatory M2 phenotype in MCAO mice in vivo and post OGD/R in vitro. Besides, PPI promoted autophagy via suppressing Akt/mTOR signaling in microglia, while inhibition of autophagy abrogated the effect of PPI on M2 microglial polarization after OGD/R. Furthermore, PPI facilitated autophagy-mediated ROS clearance to inhibit NLRP3 inflammasome activation in microglia, and NLRP3 inflammasome reactivation by nigericin abolished the effect of PPI on M2 microglia polarization. In conclusion, PPI alleviated post-stroke neuroinflammation and tissue damage via increasing autophagy-mediated M2 microglial polarization. Our data suggested that PPI had potential for ischemic stroke treatment.

**Supplementary Information:**

The online version contains supplementary material available at 10.1186/s10020-024-00828-5.

## Introduction

Ischemic stroke can be induced by thrombosis or embolism, which lead to a blockage in blood flow. Ischemic stroke ranks the third leading cause of death and a major cause for disability worldwide (Jurcau and Simion 2021). Ischemic stroke may affect 15 million people every year. Among them, 5 million of them will die and the other 5 million are permanently disabled (Maida et al. 2020). A number of risk factors are involved in the pathogenesis of ischemic stroke, such as smoking, hypertension, diabetes, and hyperlipidemia (Kleindorfer et al. 2021). Cerebral ischemia is a common form of ischemic stroke and representing 87% of all stroke cases (Krishnamurthi et al. 2013). During cerebral ischemia, an interruption of cerebral blood flow occurs, which leads to severe damage to part of the brain due to low oxygen and lack of nutrients. Reestablishing the blood flow by recanalization strategies is the key point for cerebral ischemia treatment. Nevertheless, this may cause further damage to brain tissues and trigger a series of pathological cascades, including oxidative stress, inflammatory response, disruption of blood brain barrier, and cerebral edema (Lin et al. 2016). Following cerebral ischemic stroke, there is secondary neuroinflammation, which can both induce brain damage and promote tissue recovery. Neuroinflammation may lead to the death of neuron cells, breaking of the blood barrier and hemorrhagic transformation in the early phase of cerebral ischemia. Neuroinflammation can boost the cells in brain and increase the infiltration of numerous inflammatory cytotypes in the area of ischemia, and finally supports tissue repair (Chamorro and Hallenbeck 2006). Besides, neuroinflammation is a significant controllable factor. Thus, it is important to clarify the underlying molecular mechanism of neuroinflammation and develop novel strategies to combat these pathological processes.

Microglia are tissue-resident macrophages in the nervous system and act vital role in brain development, neural environment maintenance and inflammatory response to brain injury (Orihuela et al. 2016). Microglia account for as many as 10–15% of the total cell population in the brain parenchyma (Carson et al. 2006). During cerebral ischemia, microglia can induce a robust and persistent inflammatory response throughout the progression of disease. Accumulated evidences demonstrate that microglia play an important role in cerebral ischemia injury and recovery (Wang et al. 2023a). Like peripheral macrophages, microglia are highly plastic and may rapidly polarize to either pro-inflammatory M1 phenotype or anti-inflammatory M2 phenotype in respond to environmental stimuli (Wang et al. 2023a). M1 microglia secrete pro-inflammatory cytokines such as TNF-α, IL-1β and IL-6 to exacerbate inflammation and tissue damage, while M2 microglia produce anti-inflammatory cytokines like TGF-β, IL-4 and IL-13 to alleviate inflammation and promote tissue recovery (Xiong et al. 2016). There are many studies prove that the dynamic changes between M1 and M2 type microglia are critical for controlling the inflammatory response and facilitate tissue repair in cerebral ischemia (Kanazawa et al. 2017). Furthermore, the two polarization types often overlap and may transform to each other by both intracellular or extracellular stimuli. Among them, metabolic reprogramming induced by autophagy plays crucial role in microglial polarization (Zubova et al. 2022). For example, sestrin2 promotes M1 to M2 microglia polarization via increasing mTOR-mediated autophagy in ischemic mouse brain, thus reduces neurological deficits, infarction volume, and cell apoptosis (He et al. 2020). In the present study, we explored the potential function of PPI on autophagy-mediated microglia polarization during cerebral ischemia/reperfusion injury.

Polyphyllin I (PPI) is a steroidal saponin isolated from traditional Chinese medicinal herb *Paris polyphylla* (also called Chonglou). Chonglou is commonly used to treat inflammation, abscess, parotitis and hemorrhage-related diseases in China (Li et al. 2023a). PPI is one of the main active ingredients of Chonglou. PPI is a bioactive phytochemical with molecular weight of 855.02 Da and chemical formula of C_44_H_70_O_16_. Emerging evidences prove that PPI has multiple bioactivities, including anti-cancer (Luo et al. 2022), anti-inflammation (Wang et al. 2018), anti-bacteria (Sun et al. 2023) and anti-oxidant (Huang et al. 2020). For example, PPI ameliorates pressure over-load induced cardiac dysfunction by suppressing Wnt/β-catenin pathway in a mice cardiac hypertrophy model (Li et al. 2020). In non-small cell lung cancer, PPI induces cancer cell apoptosis and inhibits growth by inhibiting UPR-mediated CHOP ubiquitination and degradation (Liu et al. 2021a). In hepatocellular carcinoma, PPI inhibits the formation of vasculogenic mimicry in tumor tissues via suppressing the PI3k-Akt-Twist1-VE-cadherin pathway (Xiao et al. 2018). To date, the potential function of PPI in cerebral ischemia is not evaluated yet. In our study, a mouse model of MCAO was constructed to evaluate the influence of PPI in cerebral ischemia–reperfusion injury. We found that PPI reduced infarct volume, alleviated neuroinflammation and improved functional recovery of mice after MCAO. In addition, PPI facilitated M1 to M2 microglial polarization in MCAO mice or after OGD/R. PPI promoted autophagy-mediated ROS clearance to inhibit NLRP3 inflammasome activation in microglia. Our results provided a novel role of PPI in cerebral-ischemia injury.

## Materials and methods

### Transient middle cerebral artery occlusion (MCAO) model and mice treatment

To mimic cerebral ischemia–reperfusion injury in vivo, a 60-min transient MCAO mice model was constructed as previously described (Al Mamun et al. 2020). Briefly, C57BL/6 mice were anesthetized by 1% pentobarbital sodium (100 mg/kg) intraperitoneally. Mice was kept warm by placing on a thermostatic blanket. Rectal temperature was maintained at 37.0 °C ± 0.5 °C during surgery. Then, a midline ventral neck incision was made to reveal the left common carotid artery (CCA), external carotid artery (ECA) and internal carotid artery (ICA). The CCA was ligated at the distal end by surgical nylon monofilament. The ECA was ligated at the end and near ICA and ECA bifurcations. Next, a small incision was cut between the nylon filaments of ECA. A 6.0 mm monofilament was inserted from left ECA, through the bifurcation of CCA, into the intracranial segment of ICA to block the blood flow at middle cerebral artery for 60 min. The regional cerebral blood flow (rCBF) was monitored by laser Doppler flowmetry (PeriFlux System 5000, PERIMED, Sweden). A decline of rCBF ≥ 70% was considered as successful occlusion. At the end of experiment, the monofilament was withdrawn to allow reperfusion. Mice in sham group underwent the same surgical procedures except the insertion of monofilament into ICA. After MCAO surgery, mice were kept warm on the thermostatic blanket until they woke up, and the wounds were sterilized with iodine every day for 2 days. In animal experiments, polyphyllin I (#S9114), MHY1485 (#S7811) and 3-MA (#S2767) were all purchased from Selleck Chemicals (USA) and dissolved in 1% DMSO + 5% PEG300 + 5% Tween 80 + 89% deionized water. Thus 1% DMSO + 5% PEG300 + 5% Tween 80 + 89% deionized water was used as vehicle control in animal studies. Mice were treated with 2 mg/kg, 5 mg/kg PPI, 10 mg/kg MHY1485, 10 mg/kg 3-MA or equal volume of vehicle control daily as indicated for one week immediately after MCAO surgery or 24 h before MCAO surgery. Mice in sham group also treated with vehicle at the same time.

### TTC (2,3,5‑triphenyltetrazolium chloride) staining

TTC staining was performed to evaluate the infarct volume of mice brain. Briefly, brains were embedded in optimal cutting temperature compound (OTC) and frozen at -20℃. Then, cut the brain into 2 mm coronal sections and stained with 2% TTC dye reagent at 37 °C for 20 min avoiding light. The sections were then washed with PBS and fixed with 4% paraformaldehyde at 4 °C overnight. Infarct volume of each section was calculated by photoshop software. Infarct area was the white region, and the normal tissue was the dark red region. Infarct size was calculated as follows: infarct size (%) = (contralateral area − ipsilateral non-infarct area)/contralateral area × 100%.

### Hematoxylin and eosin (H&E) staining

Mice brain was fixed by cardiac perfusion with 4% paraformaldehyde for 10 min. Then, the mice were decapitated, and brain tissues were fixed by 4% paraformaldehyde at 4 °C overnight. Next, the brain tissues were embedded by paraffin and cut into 8 μm sections. The sections were stained with H&E (Beyotime, China) according to manufacturer’s instructions. Neuronal damage was evaluated by denatured cell index: degenerated cells/ total number of cells.

### TUNEL staining

Apoptotic cells in mice brain were detected by TUNEL staining kit (Beyotime, China) as protocol indicated. Briefly, paraffin-embedded brain sections were dewaxed. Then, sections were digested with 2% proteinase K for 30 min at 37 °C and washed with PBS for twice. Next, the sections were incubated in TUNEL mixture at 37 °C for 30 min, and washed with PBS for three times. Sections were revealed by DAB reagents. Images were obtained by microscope (Nikon, Japan).

### Reverse transcription–quantitative PCR (RT-qPCR)

Total RNA was extracted using TRIzol reagent (Takara, Japan) according to manufacturers’ instructions. RNA concentration was measured by Nanodrop (Invitrogen, USA). A total of 1 μg RNA was reverse transcribed using High Capacity cDNA Reverse Transcription Kits (Applied biosystems, USA). Real-time PCR was performed using the SYBR gene PCR Master Mix (TOYOBO, Japan) on a LightCycler 480 PCR system (Roche, Switzerland). The primers used in RT-qPCR were: TNF-α, forward, 5ʹ-ACCAC CATCA AGGAC TCA-3ʹ, reverse, 5ʹ-AGGTCTGAAGGTAGGAAGG-3ʹ; IL-1β, forward, 5ʹ-GAATC TATAC CTGTC CTGTG TA-3ʹ, reverse, 5ʹ-CTTGT GCTCT GCTTG TGA-3ʹ; IL-6, forward, 5ʹ-CAGAA GGAGT GGCTA AGGA-3ʹ, reverse, 5ʹ-CGTAG AGAAC AACAT AAGTC AG-3ʹ; MCP-1, forward, 5ʹ-CATCA CGGAC AGAGG TTC-3ʹ, reverse, 5ʹ-TTCAC TCTTG CTCAC ATCAT-3ʹ.

### Enzyme-linked immunosorbent assay (ELISA)

The production of cytokines TNF-α (Abcam# ab208348), TGF-β (Abcam# ab277719), IL-1β (Abcam# ab197742), IL-6 (Abcam# ab222503), MCP-1 (Abcam# ab208979), IL-4 (Abcam# ab242230) and IL-13 (Abcam# ab219634) in mice brain or culture supernatants of primary microglia was evaluated by ELISA assay kits (Abcam, USA) according to manufacturers’ instructions. Briefly, samples were added into antibody-coated plates, biotinylated antibodies, streptavidin antibodies, substrate solution and stop solution consecutively. Each sample had three duplicates. The optimal density at 450 nm was detected by a microplate reader.

### Neurological score

Neurological dysfunction was determined by the modified neurological severity score (mNSS) at day 1 and day 14 post MCAO surgery. The mNSS had motor tests (flexion of forelimb and hindlimb and head movement, scored 0 to 6), beam balance tests (scored 0 to 6), and reflexes absent and abnormal movement tests (scored 0 to 2). The total mNSS score of 1 to 4, 5 to 9 and 10 to 14 represented slight, moderate and serious neurological dysfunction. The mNSS score was evaluated by three independent researchers.

### Hanging wire test, rotarod test and foot-fault test

Hanging wire test was used to test the limb grasping ability. In this test, each mouse was placed on a wired housing-cage lid. Then it was quickly inverted. The time of mice to fall from the lid was measured. In rotarod test, mice were pre-trained 4 times a day for three consecutive days. The data in the training course were recorded as baseline. Then, mice were placing on the accelerated rotating rod from 0–300 rpm within 5 min. Record the time of each mouse to fall from the rod and repeated for 3 consecutive trials with a 15-min rest between each trail. In foot-fault test, mice were placed on stainless-steel grid floor. The total number of steps was counted and the number of forelimb foot-fault was recorded. Before formal test, mice were pre-trained for three times and data in the last training course were regarded as baseline.

### Open-field test and Morris water maze test

The locomotor activity of mice was determined by open-field test. In an open field cubic box (50 cm × 50 cm × 30 cm), mice were placed the same start place, then recorded their movement for 10 min. The total distance moved and mean velocity were calculated. Morris water maze (MWM) test was conducted to assess the spatial learning and memory of mice after MCAO. The MWM test was conducted in a 1.2 diameter circular tank filled with opaque water (~ 22 °C). A fixed platform was hidden 1 cm under water in the target quadrant. Mice were pre-trained 4 times a day for 6 consecutive days. In the training, mice were allowed to search for the platform for 1 min. Mice that had not found the platform in 1 min would be guided to stay on the platform for 30 s. At day 7, a probe test was performed. In the probe test, the platform was removed and mice were allowed to search for the platform for 60 s to evaluate the memory of the mice. In the formal MWM test, mice were allowed to search platform for 60 s, and the latency time to find the platform, number of cross platform and time in target quadrant were recorded.

### Flow cytometry

To evaluate immune cells in mice brain, brain tissues were cut into pieces and digested with 150 μL collagenase (1 mg/mL) and 250 μL DNase (10 mg/mL) for 1 h at 37 °C. Next, cells were harvested and passed through 70 μm strainer. Red Blood Cell Lysis Solution (Beyotime, China) was used to deplete erythrocytes. To evaluate adherent cells in culture dish, cells were digested by 0.05% trypsin for 3 min and separated as single cell suspension. For surface staining, 1 × 10^6^ cells were blocked with anti-FC receptor antibody at 4 °C for 15 min, then stained with indicated antibodies at 4 °C for 30 min. For intracellular staining, cells were fixed and permeabilized using the intracellular staining kit (ThermoFisher, USA). Flow cytometry was performed on a CytoFLEX (Beckman Coulter, USA) and analyzed using FlowJo software (Treestar, USA). The antibodies used in flow cytometry were: CD86 PE (#12-0861-83), CD206 FITC (#MA5-16870), CD11b APC (#RM2805), Iba1 (#PA5-27436), TNF-α PE-CY7 (#25-7321-82), iNOS APC (#17-5920-82), IL-10 PE (#12-7101-82), Arg1 PE-CY7 (#25-3697-82), CD4 PE (#12-0041-82), CD3 APC (#17-0038-42), CD8 PE (#MA5-17849), CD19 PE (#12-0199-42), CD20 FITC (#11-0209-42), CD56 PE (#12-0567-42), CD25 PE-CY7 (#25-0251-82) and FoxP3 PerCP-CY5.5 (#45-5773-82) were all from ThermoFisher (USA).

### Western blot

Protein lysates were extracted from brain tissues or culture cells by RIPA lysis buffer (Beyotime, China) supplemented with protease inhibitors (Sigma, USA). Protein concentration was measured by BCA kit (Beyotime, China). A total of 20 μg protein was separated by 10% SDS-PAGE and transferred onto PVDF membranes (Biorad, USA). Then, the membranes were blocked by 5% fat-free milk for 1 h at room temperature, incubated with first antibodies at 4 °C overnight and corresponding second antibodies for 1 h at room temperature. Then antibodies used in our study were: iNOS Rabbit mAb (CST#13120, 1: 1000), Arginase-1 Rabbit mAb (CST#93668, 1: 1000), GAPDH Rabbit mAb (CST#2118, 1: 1000), LC3A/B Rabbit mAb (CST#12741, 1: 1000), p62 Antibody (CST#5114, 1: 1000), Phospho-Akt (Ser473) Antibody (CST#9271, 1: 1000), Akt Antibody (CST#9272, 1: 1000), Phospho-mTOR (Ser2448) Antibody (CST#2971, 1: 1000), mTOR Rabbit mAb (CST#2983, 1: 1000), pro-IL-1β Rabbit mAb (CST#31202, 1: 1000), Cleaved-IL-1β Rabbit mAb (CST#63124, 1: 1000), pro-Caspase-3 Antibody (CST#9662, 1: 1000), Cleaved Caspase-3 Antibody (CST#9661, 1: 1000), Phospho-GSK-3α/β (Ser21/9) Antibody (CST#9331, 1: 1000), GSK-3α/β Rabbit mAb (CST#5676, 1: 1000) and NLRP3 Rabbit mAb (CST#15101, 1: 1000) were all obtained from Cell Signaling Technology (USA).

### Primary microglia isolation and OGD/R procedure

Primary microglia were separated from healthy C57BL/6 mice brain at postnatal days P1 and P2 as previously described (Fan et al. 2012). Briefly, mouse brain was dissected and cut into meninges, then passed through 70 μm cell strainer. Cells were cultured in DMEM/F12 medium (Gibco, USA) supplemented with 20% fetal bovine serum (Gibco, USA) and 1% penicillin/streptomycin at 37 °C containing 5% CO_2_. Microglia and astrocytes have different adhesive features. To separate microglia from mixed glial cultures, cells were cultured with mild agitation at 200 rpm for 6 h. The purity of adherent microglia was evaluated by flow cytometry, which indicated that more than 95% cells were Iba1 and CD11b double positive. To establish in vitro cerebral ischemia/reperfusion injury model, primary microglia were exposed to OGD/R conditions as previously described (Li et al. 2021). Briefly, microglia were incubated with prewarmed glucose-free DMEM/F12 (Gibco, USA) in an anaerobic incubator containing 5% CO_2_ and 95% N_2_ at 37 °C for 60 min. Next, cells were cultured with normal DMEM/F12 containing glucose under normoxic condition for another 24 h. For drug treatment in culture cells, PPI (#S9114), MHY1485 (#S7811), 3-MA (#S2767) and Nigericin (#S66419) were all dissolved in DMSO before use, thus DMSO was used as vehicle control in these experiments.

### Immunofluorescence staining

Primary cultured microglia were seeded on coverslips for 24 h, then fixed with 4% paraformaldehyde for 15 min at room temperature and permeabilized by 0.5% Triton X-100 for 3 min at room temperature. Thereafter, cells were blocked with 1% BSA for 15 min and incubated with LC3A/B Alexa Fluor® 488 (Cell signaling #13082, 1: 50) at 4 °C overnight. Images were captured by laser confocal microscopy (Leica, German).

### Measurement of ROS, MDA and GSH levels

Reactive oxidative species (ROS) were measured by staining with DCF-DA probe (Beyotime, China). In brief, cells were incubated with 10 μM DCF-DA at 37 °C for 20 min avoiding light, then subjected to flow cytometry analysis. Lipid peroxidation MDA assay kit (Nanjing Jiancheng#A003-4-1, China) was used to detect Malondialdehyde (MDA) concentration according to manufacturers’ instructions. GSH assay kit (Nanjing Jiancheng#A006-2-1, China) was used to evaluate GSH levels. Each sample had three duplicates.

### Statistical analysis

Data were shown as mean ± SD. GraphPad Prism 8.0 (GraphPad Software, USA) was used in statistical analysis. One-way ANOVA (Tukey's post-hoc test) or Student’s* t* test was used to evaluate difference between groups. *P* < 0.05 was considered as statistically significant.

## Results

### PPI reduces infarct volume and neuroinflammation in mice after MCAO

**Fig. 1:**
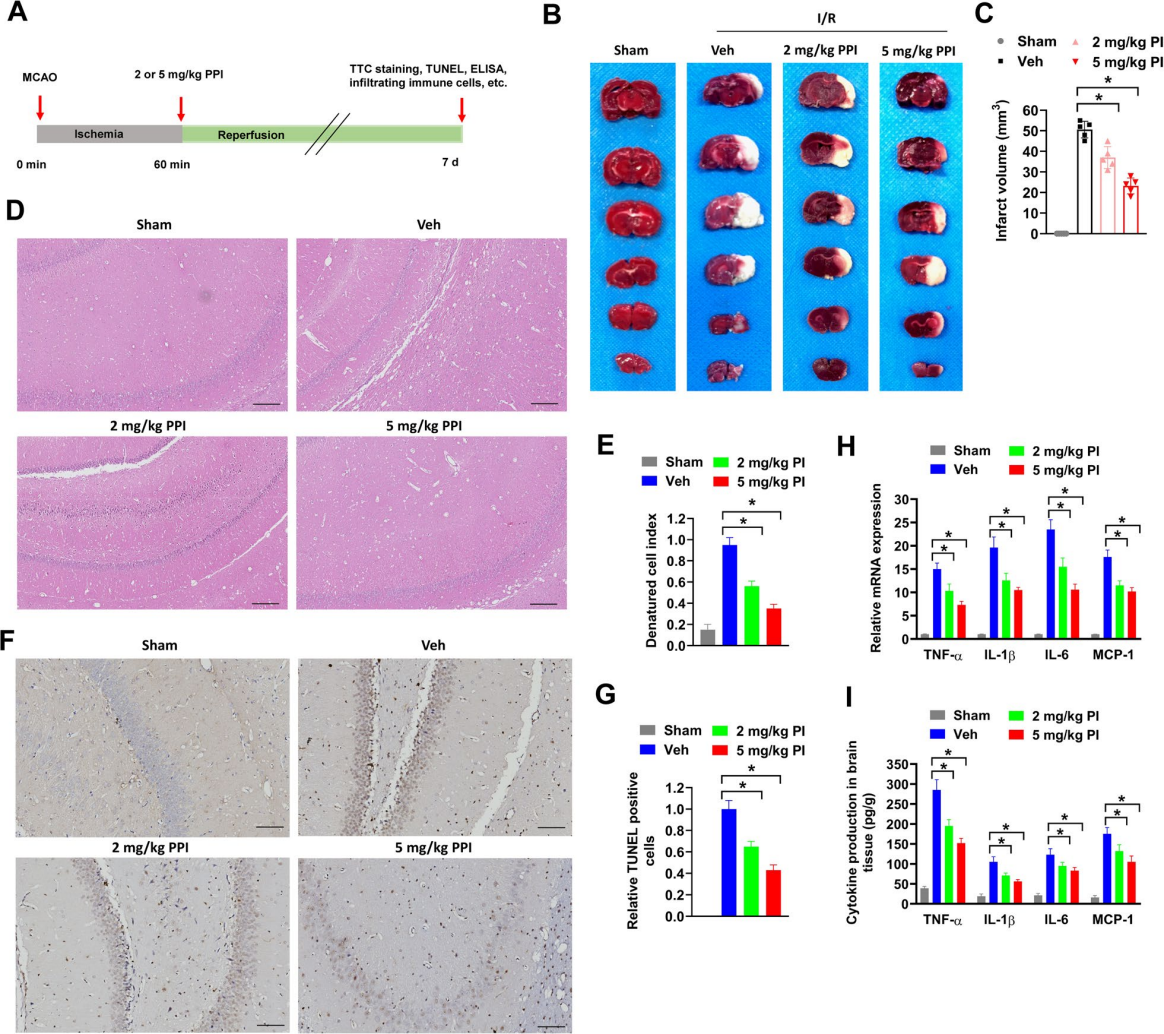
PPI alleviates cerebral ischemia–reperfusion injury and neuroinflammation in mice after MCAO. **A** the experimental design of animal studies. B-C, MCAO mice were treated with PPI or vehicle control (Veh group) daily for 7 d immediately after surgery, then infarct volume was evaluated by TTC staining. Representative images (**B**) and infarct volumes (**C**) were shown. Mice in sham group received equal volume of vehicle treatment at the same time (n = 5 for each group, *t* test). **D**, **E** histopathological changes of brain tissues were checked by H&E staining. Representative images (**D**) and denatured cell index (**E**) were shown (n = 5 for each group, *t* test). **F**, **G** apoptotic cells in mice brain were evaluated by TUNEL staining. Representative images (**F**) and relative TUNEL positive cells (**G**) were shown (n = 5 for each group, *t* test). **H**, **I** relative mRNA expression and secretion of indicated cytokines in ischemic brain tissues were evaluated by RT-qPCR (**H**) and ELISA assay (**I**) (n = 5 for each group, *t* test). *P < 0.05

To evaluate the potential influence of PPI on cerebral ischemia–reperfusion (I/R) injury and post-stroke recovery, a mouse model of transient MCAO was established. Firstly, the potential toxicity of PPI on C57BL/6 mice were evaluated. We found that 2 or 5 mg/kg PPI treatment daily for 4 weeks showed no obvious influence on body weight of mice compared with vehicle control (Supplementary Fig.  1A). Besides, the dosages of 1 to 5 mg/kg PPI were commonly used in animal experiments (He et al. 2019; Chang et al. 2017; Zheng et al. 2023). In our study, mice were immediately treated with 2 or 5 mg/kg PPI for 7 days after reperfusion (Fig. 1A). Infarct volume was evaluated by TTC staining. Compared with MCAO mice treated with vehicle control (Veh group), MCAO mice treated with 2 or 5 mg/kg PPI (2 mg/kg PPI and 5 mg/kg PPI group) showed reduced infarct volume, suggesting that PPI had protective effects on cerebral ischemia–reperfusion injury (Fig. 1B and C). IHC staining was used to evaluate the histopathological changes of the infarct brain. Compared with Sham group, MCAO mice in Veh group showed severe tissue damage in the hippocampus region of brain, with apparently damaged neuron structure, neuronal loss and numerous vacuolated spaces (Fig. 1D and E). However, PPI treatment evidently alleviated these pathological changes (Fig. 1D and E). In addition, cell apoptosis in the hippocampus region of brain was examined by TUNEL staining. The number of TUNEL positive cells were significantly increased in MCAO mice of Veh group compared with Sham group, but these effects were diminished MCAO mice of 2 mg/kg PPI and 5 mg/kg PPI group (Fig. 1F and G). Next, neuroinflammation in ischemic brain was evaluated. Compared with mice in Sham group, the mRNA expression and secretion of pro-inflammatory cytokines (TNF-α, IL-1β, IL-6 and MCP-1) were significantly increased in brain tissues of MCAO mice in Veh group (Fig. 1H and I). In comparison, PPI treatment obviously reduced the mRNA expression and secretion of these pro-inflammatory cytokines compared with MCAO mice in Veh group (Fig. 1H and I). As ischemia–reperfusion injury is quick and enormous ROS are generated immediately, we had treated MCAO mice with PPI one day before surgery and lasted for 7 d in order to observe the actual effect of PPI in ischemia–reperfusion injury. As expected, PPI treatment one day before MCAO surgery reduced brain damage, cell death and neuroinflammation as the same (Supplementary Figure S1B-1G). Taken together, PPI alleviated cerebral ischemia–reperfusion injury and neuroinflammation in mice after MCAO.

### PPI improves functional recovery of MCAO mice

**Fig. 2:**
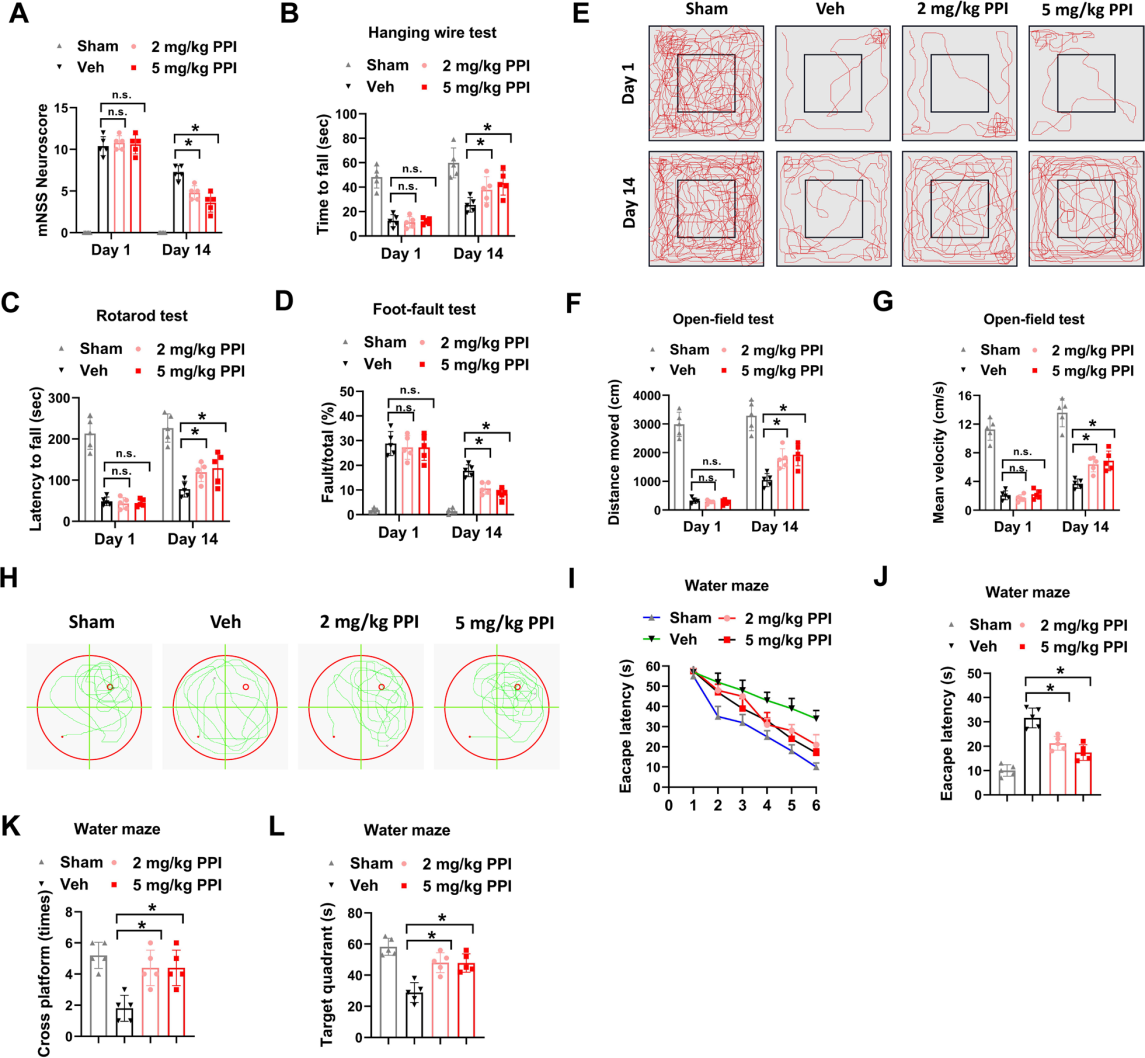
PPI improves functional recovery of MCAO mice. **A** neurological dysfunction of MCAO mice treated with PPI or vehicle control was evaluated by mNSS at day 1 and day 14 post-MCAO (n = 5 for each group, *t* test). **B**–**D**, the locomotor deficits of MCAO mice were evaluated at day 1 and day 14 post-MCAO. The time to fall (**B**) in hanging wire test, latency to fall (**C**) in rotarod test, and percentage of fault/total in foot-fault test (**D**) were shown (n = 5 for each group, *t* test). **E**–**G**, the behavior improvement was evaluated by open-field test at day 1 and day 14 post-MCAO. The representative motion trajectory (**E**), distance moved (**F**) and mean velocity (**G**) were shown (n = 5 for each group, *t* test). **H**–**L** the spatial learning and memory functions of MCAO mice was detected by Morris water maze test at day 14 post-MCAO. Representative swimming traces (**H**), time to escape (I and J), times of crossing platform (**K**), and time in target quadrant (**L**) were shown (n = 5 for each group, *t* test). *P < 0.05

The influence of PPI on poststroke functional recovery of MCAO mice was evaluated. Neurological deficits were assessed by a modified neurological severity score (mNSS). Compared with mice in Sham group, mice in MCAO groups had high mNSS score (Fig. 2A). Meanwhile, there was no apparent difference of mNSS score between mice in Veh group, 2 mg/kg PPI or 5 mg/kg PPI group at day 1 (Fig. 2A). However, the mNSS score of mice in 2 mg/kg PPI and 5 mg/kg PPI group was much lower than mice in Veh group at day 14 poststroke (Fig. 2A). In hanging wire test and rotarod test, mice in MCAO groups had a shorter time to fall compared with mice in Sham group at day 14 poststroke, indicating impaired strength and motor coordination caused by cerebral ischemia (Fig. 2B and C). Nevertheless, these locomotor deficits of MCAO mice were evidently ameliorated by 2 mg/kg PPI or 5 mg/kg PPI treatment (Fig. 2B and C). In foot-fault test, PPI treatment also reduced the number of contralateral forelimb foot-faults (Fig. 2D). The behavior improvement was evaluated by open-field test. MCAO mice in Veh group and PPI groups showed reduced spontaneous exploration behavior, distance moved and mean velocity compared with mice in Sham group at day 1 post-stroke (Fig. 2E–G). Furthermore, MCAO mice in 2 mg/kg PPI and 5 mg/kg PPI treatment groups displayed increased spontaneous exploration behavior, distance moved and mean velocity compared with MCAO mice in Veh group at day 14 poststroke (Fig. 2E–G). The spatial learning and memory functions of MCAO mice was detected by Morris water maze test. MCAO mice in Veh group showed significant cognitive deficits compared with mice in Sham group, including latency to find the submerged platform, decreased number of platform crossovers and less time in the quadrant (Fig. 2H–L). In comparison, these cognitive deficits were evidently relieved by 2 mg/kg PPI and 5 mg/kg PPI treatment (Fig. 2H–L). Collectively, these data indicated that PPI improved functional recovery of MCAO mice.

### PPI modulates microglial polarization towards anti-inflammatory M2 phenotype in MCAO mice

**Fig. 3:**
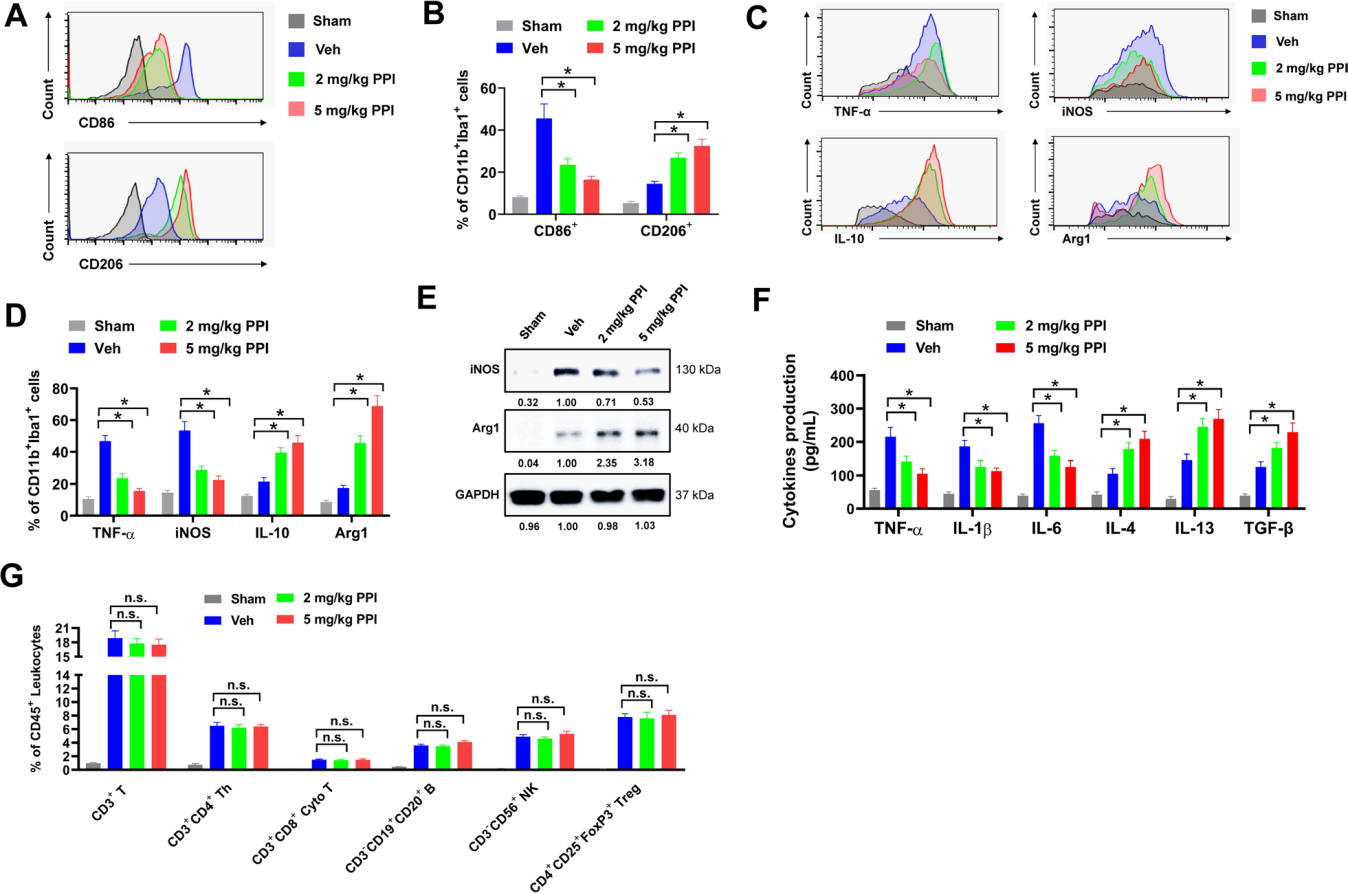
PPI induces M1-M2 phenotype shift in MCAO mice. **A**, **B** microglia separated from brain tissues of MCAO mice were evaluated by flow cytometry. Representative histogram (**A**) and percentage of CD86^+^ and CD206^+^ cells in CD11b^+^Iba1^+^ microglia (**B**) were shown (n = 5 for each group, *t* test). **C**, **D** the expression of pro-inflammatory M1 phenotype markers (TNF-α and iNOS) and anti-inflammatory M2 phenotype markers (IL-10 and Arg1) in microglia separated from brain tissues of MCAO mice were evaluated by flow cytometry. Representative histogram (**C**) and percentage of indicated genes in CD11b^+^Iba1^+^ microglia (**D**) were shown (n = 5 for each group, *t* test). **E** the protein expression of iNOS and Arg1 in microglia separated from brain tissues of MCAO mice was evaluated by western blot (n = 3 for each group, *t* test). **F** the secretion of pro-inflammatory cytokines (TNF-α, IL-1β, IL-6) and anti-inflammatory cytokines (IL-4, IL-13 and TGF-β) in culture medium of microglia separated from ischemic brain of MCAO mice was evaluated by ELISA assay (n = 3 for each group, *t* test). **G** infiltrating of T cells (CD45^+^CD3^+^), CD4^+^ T helper cells (Th, CD3^+^CD4^+^), CD8^+^ cytotoxic T cells (Cyto T, CD3^+^CD8^+^), B cells (CD3^−^CD19^+^CD20^+^), Natural killer cells (NK, CD45^+^CD3^−^NK1.1^+^) and Treg cells (CD4^+^CD25^+^FoxP3^+^) in ischemic brain of MCAO mice was evaluated by flow cytometry (n = 5 for each group, *t* test). *P < 0.05

Microglia, the major innate immune cells in the brain, can respond rapidly to ischemic injury. The balance between M1 and M2 microglia polarization plays an important role in regulating the neuroinflammatory response. To evaluate the effect of PPI on microglial polarization, cells were separated from the ischemic brain and subjected for flow cytometry. CD86 and CD206 are differential markers for M1 and M2 microglia, respectively. Iba1 and CD11b are commonly used markers for microglia. In the present study, M1 microglia were marked as CD11b^+^Iba1^+^CD86^+^, while M2 microglia were marked as CD11b^+^Iba1^+^CD206^+^. Both CD11b^+^Iba1^+^CD86^+^ M1 microglia and CD11b^+^Iba1^+^CD206^+^ M2 microglia were increased in ischemic brain of MCAO mice compared with these in Sham control, indicating that microglia were activated after MCAO surgery (Fig. 3A and B). However, the percentages of CD11b^+^Iba1^+^CD206^+^ M2 microglia were evidently increased while CD11b^+^Iba1^+^CD86^+^ M1 microglia decreased by 2 mg/kg and 5 mg/kg PPI treatment, suggesting that PPI promoted M1 to M2 microglia transition (Fig. 3A and B). M1 microglia are pro-inflammatory, and M2 microglia are anti-inflammatory. The expression levels of pro-inflammatory M1 phenotype markers (TNF-α and iNOS) and anti-inflammatory M2 phenotype markers (IL-10 and Arg1) were evaluated by flow cytometry. The percentages of TNF-α^+^ and iNOS^+^ microglia were evidently decreased while IL-10^+^ and Arg1^+^ microglia increased by 2 mg/kg and 5 mg/kg PPI treatment in ischemic brain of MCAO mice compared with these in Veh group (Fig. 3C and D). This was also validated by western blot and ELISA assay. Microglia from the ischemic brain of MCAO mice were isolated by flow cytometry. In our study, the protein expression of Arg-1 was increased while the protein expression of iNOS reduced in microglia separated from the ischemic brain of MCAO mice in 2 mg/kg and 5 mg/kg PPI treatment groups compared with these in Veh group (Fig. 3E). In ELISA assay, the secretion of pro-inflammatory cytokines (TNF-α, IL-1β, IL-6) was obviously reduced while anti-inflammatory cytokines (IL-4, IL-13 and TGF-β) increased in culture medium of microglia separated from ischemic brain of MCAO mice in 2 mg/kg and 5 mg/kg PPI treatment groups compared with these in Veh group (Fig. 3F). Besides, the infiltrating of other immune cells in ischemic brain of MCAO mice was further evaluated. However, PPI treatment showed no apparent influence on the infiltrating of T cells (CD45^+^CD3^+^), CD4^+^ T helper cells (Th, CD3^+^CD4^+^), CD8^+^ cytotoxic T cells (Cyto T, CD3^+^CD8^+^), B cells (CD3^−^CD19^+^CD20^+^), Natural killer cells (NK, CD45^+^CD3^−^NK1.1^+^) and Treg cells (CD4^+^CD25^+^FoxP3^+^) in ischemic brain of MCAO mice (Fig. 3G). Above all, these results indicated that PPI modulated microglial polarization towards anti-inflammatory M2 phenotype in MCAO mice.

### PPI facilitates M1 to M2 microglial transition in vitro after OGD/R

**Fig. 4:**
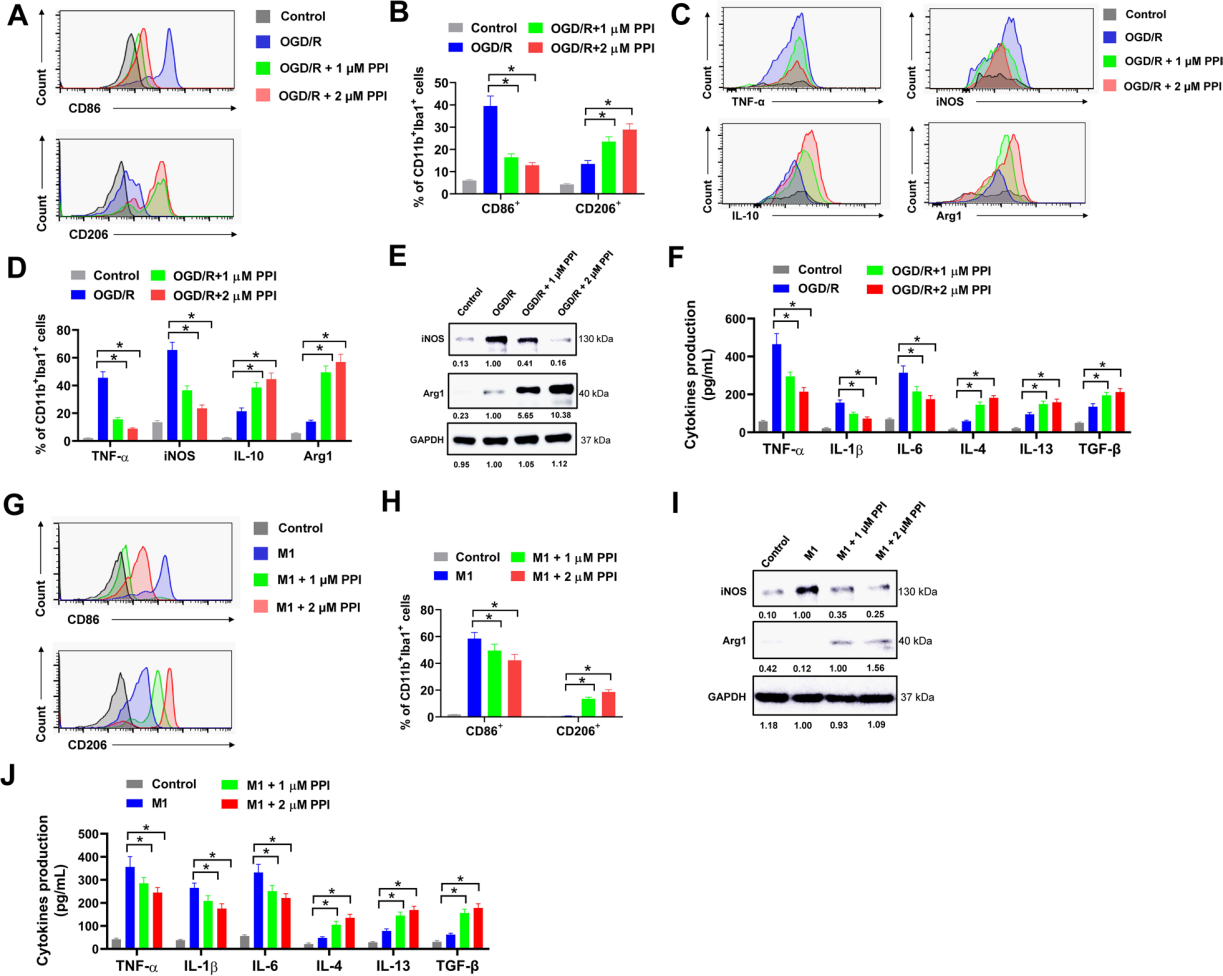
PPI promotes M1 to M2 microglial transition in vitro after OGD/R. **A**, **B** primary microglia isolated from healthy mice brain were treated with PPI (OGD/R + 1 μM PPI group and OGD/R + 2 μM PPI group) or equal volume of DMSO (OGD/R group) for 72 h post OGD/R, then evaluated by flow cytometry. Representative histogram (**A**) and percentage of CD86^+^ and CD206^+^ cells in CD11b^+^Iba1^+^ microglia (**B**) were shown (n = 3 for each group, *t* test). Cells in control group did not undergo OGD/R treatment but received equal volume of DMSO. **C**, **D** the expression of pro-inflammatory M1 phenotype markers (TNF-α and iNOS) and anti-inflammatory M2 phenotype markers (IL-10 and Arg1) in microglia post OGD/R were evaluated by flow cytometry (n = 3 for each group, *t* test). Representative histogram (**C**) and percentage of indicated genes in CD11b^+^Iba1^+^ microglia (**D**) were shown. **E** the protein expression of iNOS and Arg1 in microglia post OGD/R was evaluated by western blot (n = 3 for each group, *t* test). **F** the secretion of pro-inflammatory cytokines (TNF-α, IL-1β, IL-6) and anti-inflammatory cytokines (IL-4, IL-13 and TGF-β) in culture medium of microglia after OGD/R was evaluated by ELISA assay (n = 3 for each group, *t* test). **G**, **H** LPS and IFN-γ induced M1 microglia were treated with PPI (M1 + 1 μM PPI group and M1 + 2 μM PPI group) or equal volume of DMSO (M1 group) for 72 h, then evaluated by flow cytometry (n = 3 for each group, *t* test). Representative histogram (**G**) and percentage of CD86^+^ and CD206^+^ cells in CD11b^+^Iba1^+^ microglia (**H**) were shown. Cells in control group did not undergo LPS and IFN-γ induced M1 microglial polarization but received equal volume of DMSO at the same time. **I** the protein expression of iNOS and Arg1 in these microglia was evaluated by western blot (n = 3 for each group, *t* test). **J** the secretion of indicated cytokines in culture medium of these microglia was evaluated by ELISA assay (n = 3 for each group, *t* test). *P < 0.05

To further confirm the effect of PPI on microglial polarization, an in vitro OGD/R cell model was established to mimic cerebral ischemia–reperfusion injury. Briefly, microglia were isolated from healthy mice brain, then exposed to oxygen–glucose deprivation and reoxygenation (OGD/R) as previously described (Cai et al. 2019). In the present study, the percentage of CD11b^+^Iba1^+^CD86^+^ M1 microglia was significantly increased after OGD/R (OGD/R group) compared with non-treated control (Control group) (Fig. 4A and B). Meanwhile, PPI treatment (OGD/R + 1 μM PPI group and OGD/R + 2 μM PPI group) promoted M1 to M2 microglia shift by increasing the percentage of CD11b^+^Iba1^+^CD206^+^ M2 microglia and decreasing the percentage of CD11b^+^Iba1^+^CD86^+^ M1 microglia compared with the microglia treated with vehicle control (OGD/R group) (Fig. 4A and B). Moreover, the percentages of TNF-α^+^ and iNOS^+^ microglia were obviously decreased while IL-10^+^ and Arg1^+^ microglia increased in OGD/R + 1 μM PPI group and OGD/R + 2 μM PPI group compared with these in vehicle-treated OGD/R group (Fig. 4C and D). PPI treatment also reduced the protein expression of iNOS and elevated the protein expression of Arg-1 in microglia after OGD/R treatment (Fig. 4E). Besides, PPI treatment reduced the secretion of pro-inflammatory cytokines (TNF-α, IL-1β, IL-6) and increased the secretion of anti-inflammatory cytokines (IL-4, IL-13 and TGF-β) in microglia after OGD/R treatment (Fig. 4F). Next, microglia from healthy mice brain were polarized to M1 microglia by treating with 100 ng/mL LPS and 20 ng/mL IFN-γ for 24 h in vitro. LPS and IFN-γ treatment significantly increased the percentage of CD11b^+^Iba1^+^CD86^+^ M1 microglia (M1 group) compared with that in non-treated control (Control group) (Fig. 4G and H). However, PPI treatment attenuated LPS and IFN-γ induced M1 microglial polarization and promoted M2 microglial polarization, as the percentage of CD11b^+^Iba1^+^CD86^+^ M1 microglia were decreased while CD11b^+^Iba1^+^CD206^+^ M2 microglia increased in PPI treatment groups (M1 + 1 μM PPI and M1 + 2 μM PPI groups) (Fig. 4G and H). In western blot, M1 microglia induced by LPS and IFN-γ showed increased iNOS expression, but this was weakened by PPI treatment (Fig. 4I). On the contrary, PPI treatment increased the protein expression of Arg-1 (Fig. 4I). Moreover, PPI treatment reduced the secretion of pro-inflammatory cytokines (TNF-α, IL-1β, IL-6) and increased the secretion of anti-inflammatory cytokines (IL-4, IL-13 and TGF-β) in microglia (Fig. 4J). Taken together, these results indicated that PPI promoted M1 to M2 microglial transition in vitro after OGD/R.

### PPI promotes autophagy via suppressing Akt/mTOR signaling in microglia

**Fig. 5:**
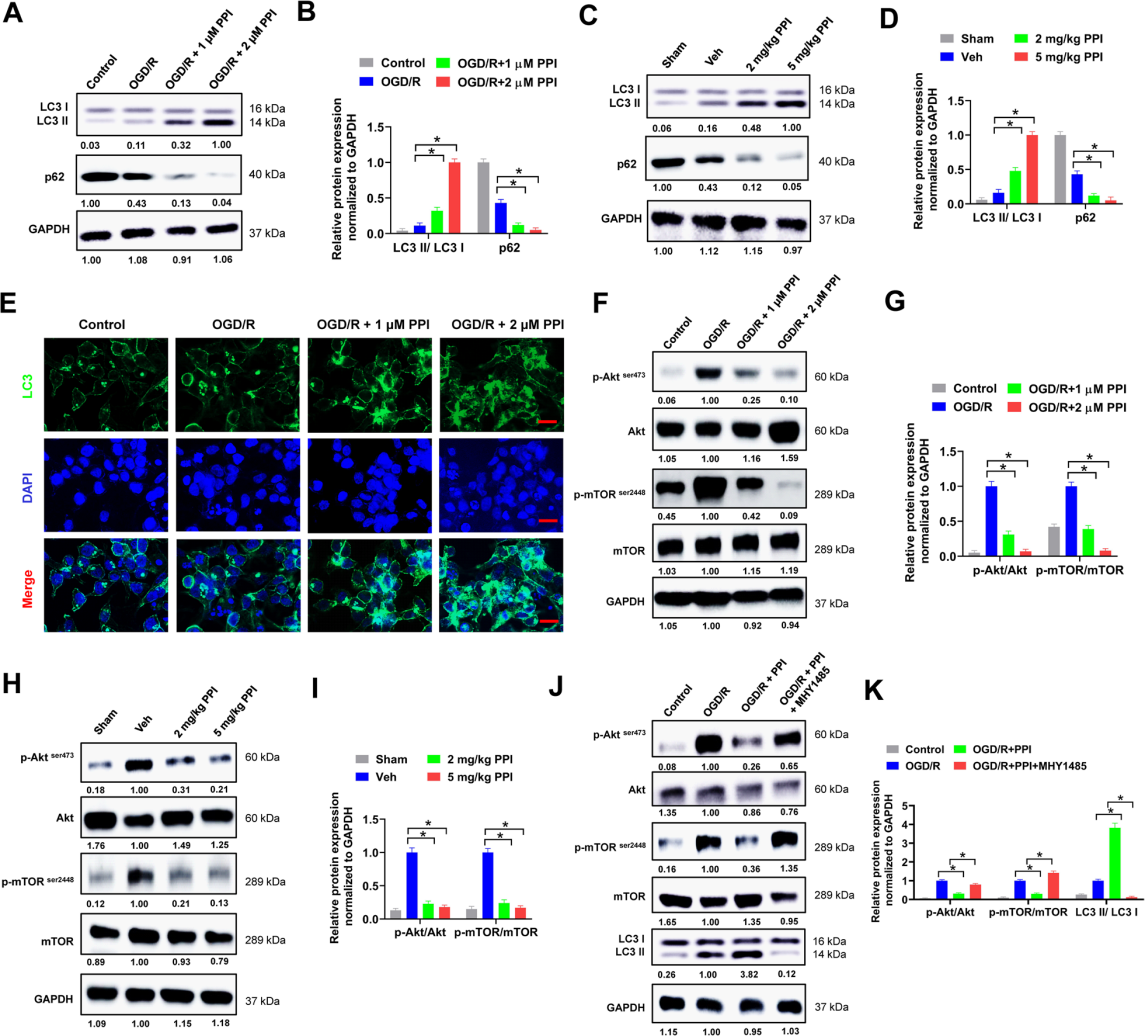
PPI induces autophagy in microglia via inhibiting Akt/mTOR signaling. **A**, **B** primary microglia isolated from healthy mice brain were treated with PPI or equal volume of DMSO for 24 h post OGD/R, then collected lysates for western blot (**A**) (n = 3 for each group, *t* test). Relative protein expression of indicated genes was shown (**B**). Cells in control group did not undergo OGD/R treatment but received equal volume of DMSO. **C**, **D** MCAO mice were treated with PPI or equal volume of vehicle reagents (Veh group) daily for 7 d immediately after surgery, then microglia were separated from ischemic brain and collected lysates for western blot (**C**). Relative protein expression of indicated genes was shown (**D**) (n = 3 for each group, *t* test). **E** the protein expression of LC3 in primary microglia isolated from healthy mice brain was evaluated by immunofluorescence. **F**–**I** the protein expression of p-Akt, Akt, p-mTOR and mTOR in microglia after OGD/R treatment (**F**, **G**) or separated from MCAO mice (**H**, **I**) was evaluated by western blot (n = 3 for each group, *t* test). The ratio of p-Akt/Akt and p-mTOR/mTOR (**G** and **I**) was shown. **J**, **K** primary microglia isolated from healthy mice brain were treated with 2 μM PPI, 10 μM MHY1485 or equal volume of DMSO as indicated post OGD/R, then collected lysates for western blot (**J**). The ratio of p-Akt/Akt and p-mTOR/mTOR (**K**) was shown (n = 3 for each group, *t* test). *P < 0.05

There are increasing evidences demonstrating that autophagy is involved in microglial polarization (Zubova et al. 2022). Moreover, recent studies prove that PPI can induce autophagy in various diseases (Luo et al. 2022; He et al. 2019; Li et al. 2023b). Thus, we speculated that PPI might promote M2 microglial polarization via regulating autophagy. Conversion of LC3 I to LC3 II is a vital step for autophagy, while p62 is a multifunctional adapter protein implicated in selective autophagy (Klionsky et al. 2021). In the present study, PPI treatment obviously promoted LC3 I to LC3 II conversion and decreased p62 expression in microglia after OGD/R, indicating that PPI induced autophagy in microglia (Fig. 5A and B). This was further validated in MCAO mice. PPI treatment facilitated LC3 I to LC3 II conversion and suppressed p62 expression in microglia isolated from ischemic brain of MCAO mice (Fig. 5C and D). The influence of PPI on autophagy was also evaluated by immunofluorescence. In our study, PPI treatment resulted in a large number of LC3 puncta in microglia, suggesting that PPI treatment promoted the formation of autophagosomes (Fig. 5E). To explore the potential pathway involved in PPI-induced autophagy, we focused on Akt/mTOR signaling, which is the central regulator of autophagy by modulating multiple aspects of the autophagy process (Zhu et al. 2019a). In our study, we found that PPI treatment apparently reduced the phosphorylation of Akt and mTOR in microglia after OGD/R treatment (Fig. 5F and G) or from ischemic brain of MCAO mice (Fig. 5H and I), indicating that PPI suppressed Akt/mTOR signaling in microglia. In addition, previous studies indicate that GSK-3 isoforms (GSK-3α and GSK-3β) are downstream targets of Akt and regulate mTOR and autophagy (Ryu et al. 2021; Zhou et al. 2013). However, we found that PPI treatment showed no apparently influence on the total and phosphorylation of GSK-3α and GSK-3β in microglia after OGD/R treatment (Supplementary Figure S2A and S2B) or from ischemic brain of MCAO mice (Supplementary Figure S2C and S2D). MHY1485 is a commonly used mTOR activator. Co-treatment with MHY1485 abrogated the inhibitory effect of PPI on Akt/mTOR signaling in microglia after OGD/R treatment (Fig. 5J and K). Furthermore, MHY1485 treatment reversed the influence of PPI on LC3 I to LC3 II conversion and p62 expression, suggesting that MHY1485 suppressed PPI-induced autophagy (Fig. 5J and K). Collectively, the above results suggested that PPI promoted autophagy via suppressing Akt/mTOR signaling in microglia.

### Inhibition of autophagy abrogates the effect of PPI on M2 microglial polarization

**Fig. 6:**
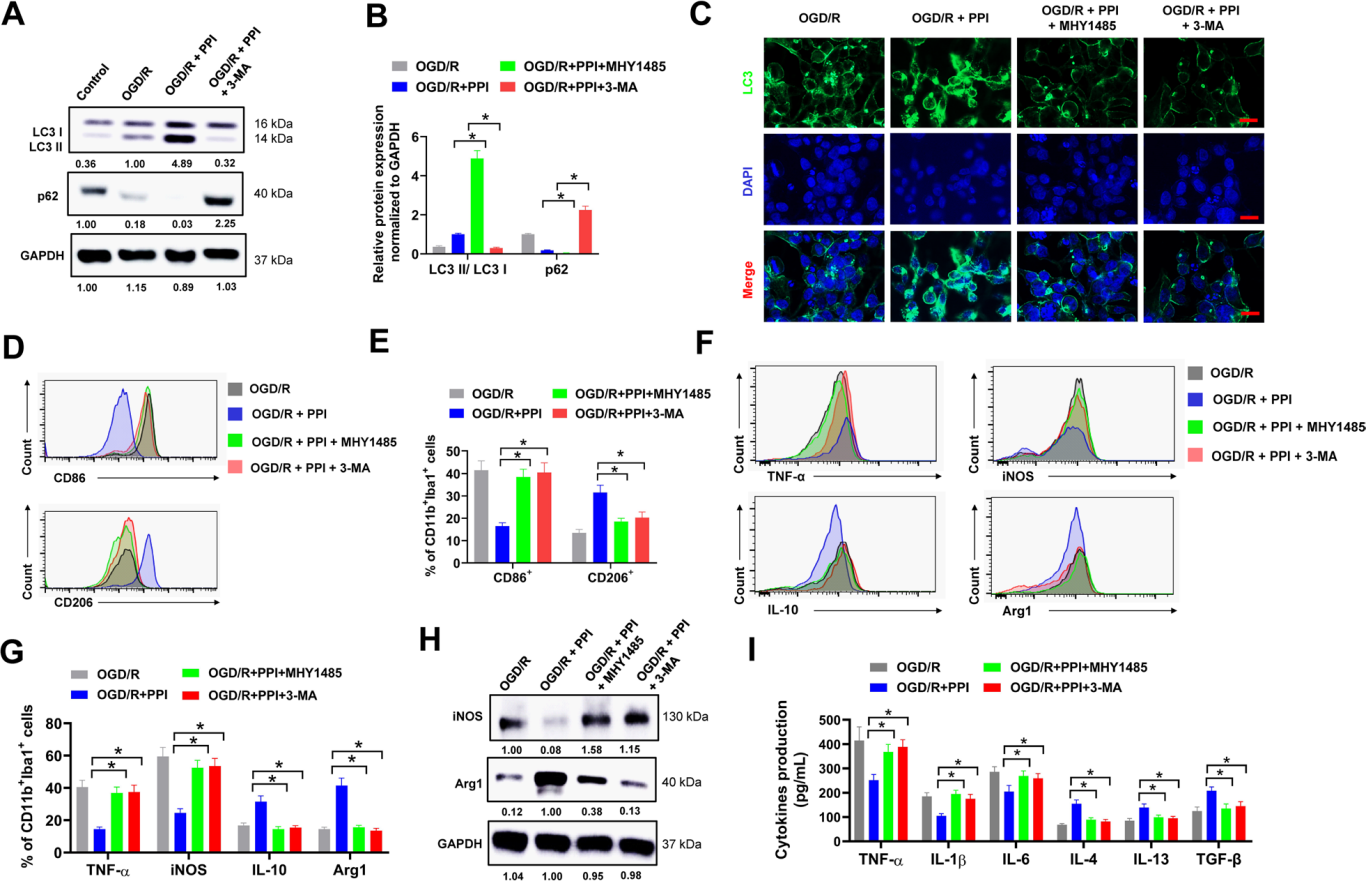
Inhibition of autophagy abrogates the effect of PPI on M2 microglial polarization. **A**, **B** primary microglia isolated from healthy mice brain were treated with 2 μM PPI, 5 mM 3-MA or equal volume of DMSO as indicated for 24 h post OGD/R, then collected lysates for western blot (**A**). Relative protein expression (**B**) was shown (n = 3 for each group, *t* test). **C** the protein expression of LC3 in primary microglia treated as indicated was evaluated by immunofluorescence. **D**, **E** primary microglia isolated from healthy mice brain were treated with 2 μM PPI, 5 mM 3-MA or equal volume of DMSO as indicated for 72 h post OGD/R, then evaluated by flow cytometry (n = 3 for each group, *t* test). Representative histogram (**D**) and percentage of CD86^+^ and CD206^+^ cells in CD11b^+^Iba1^+^ microglia (**E**) were shown. **F**, **G** the expression of pro-inflammatory M1 phenotype markers (TNF-α and iNOS) and anti-inflammatory M2 phenotype markers (IL-10 and Arg1) in microglia post OGD/R were evaluated by flow cytometry. Representative histogram (**F**) and percentage of indicated genes in CD11b^+^Iba1^+^ microglia (**G**) were shown (n = 3 for each group, *t* test). **H** the protein expression of iNOS and Arg1 in microglia post OGD/R was evaluated by western blot (n = 3 for each group, *t* test). **I** the secretion of pro-inflammatory cytokines (TNF-α, IL-1β, IL-6) and anti-inflammatory cytokines (IL-4, IL-13 and TGF-β) in culture medium of microglia after OGD/R was evaluated by ELISA assay (n = 3 for each group, *t* test). *P < 0.05

To investigate the role of autophagy on PPI-induced microglial polarization, we used the autophagy inhibitor 3-MA and mTOR activator MHY1485. Both MHY1485 and 3-MA suppressed LC3 I to LC3 II conversion and recovered p62 expression in PPI-treated microglia after OGD/R (Fig. 5J and K, Fig. 6A and B). This was further validated by immunofluorescence. PPI treatment resulted in a large number of LC3 puncta in microglia, but this was abandoned by MHY1485 or 3-MA (Fig. 6C). Next, the influence of MHY1485 and 3-MA on PPI-induced microglial polarization was evaluated. PPI treatment significantly increased the percentage of CD11b^+^Iba1^+^CD206^+^ M2 microglia and decreased the percentage of CD11b^+^Iba1^+^CD86^+^ M1 microglia after OGD/R, but this was abolished when co-treated with MHY1485 or 3-MA (Fig. 6D and E). PPI treatment reduced the percentages of TNF-α^+^ and iNOS^+^ microglia and increased the percentages of IL-10^+^ and Arg1^+^ microglia after OGD/R, but these effects were abrogated by MHY1485 or 3-MA treatment (Fig. 6F and G). This was also validated by western blot and ELISA assay. The protein expression of iNOS was reduced while Arg-1 increased by PPI treatment, however this was reversed by MHY1485 or 3-MA treatment (Fig. 6H). In ELISA assay, the secretion of pro-inflammatory cytokines (TNF-α, IL-1β, IL-6) was obviously reduced while anti-inflammatory cytokines (IL-4, IL-13 and TGF-β) increased in culture medium of microglia by PPI treatment after OGD/R, but these effects were evidently attenuated when co-treated with MHY1485 or 3-MA (Fig. 6I). Above all, our results indicated that inhibition of autophagy abrogated the effect of PPI on M2 microglial polarization after OGD/R.

### PPI facilitates autophagy-mediated ROS clearance to inhibit NLRP3 inflammasome activation in microglia

**Fig. 7:**
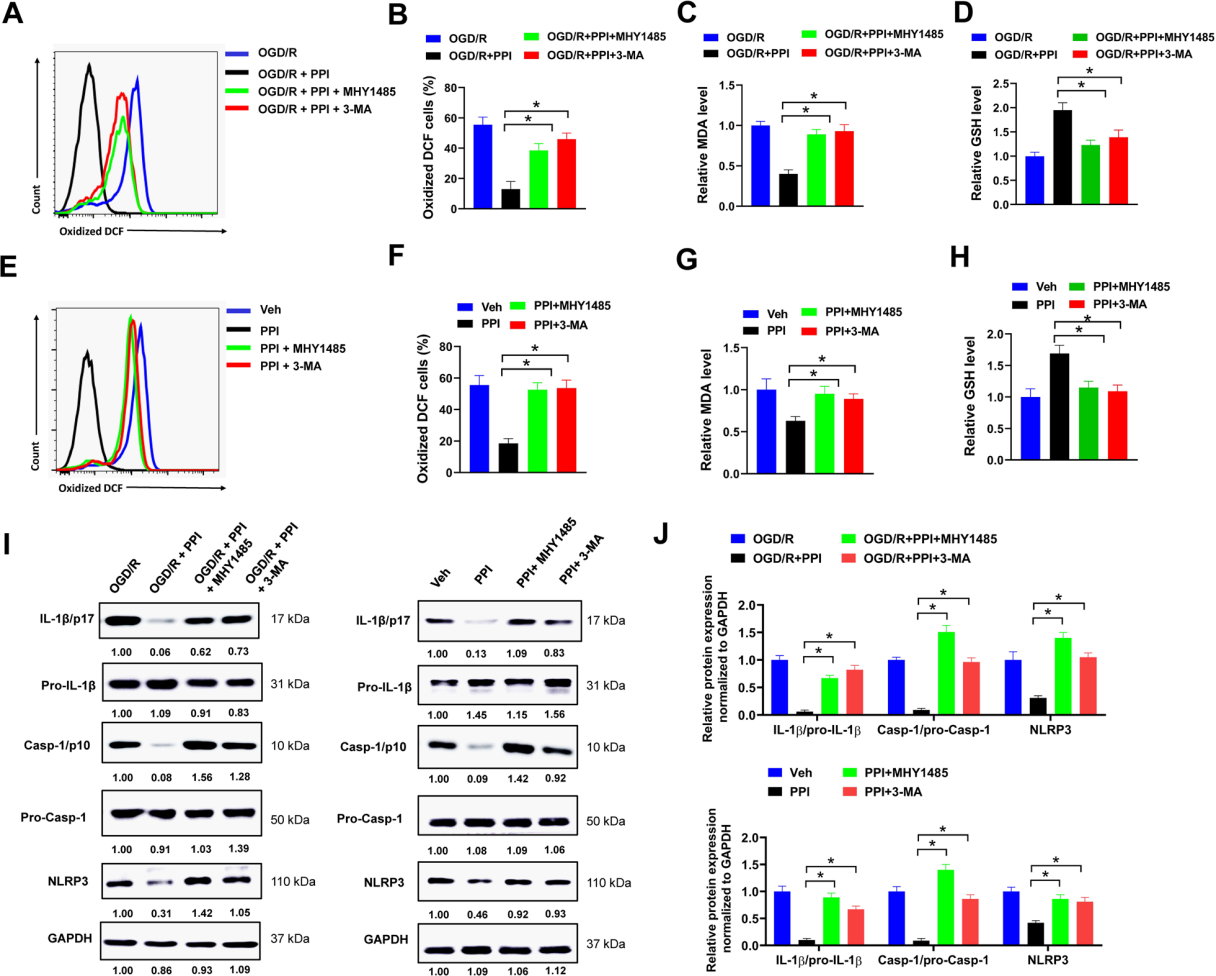
PPI facilitates autophagy-mediated ROS clearance to inhibit NLRP3 inflammasome activation in microglia. **A** primary microglia isolated from healthy mice brain were treated with 2 μM PPI, 10 μM MHY1485, 5 mM 3-MA or equal volume of DMSO as indicated for 24 h post OGD/R, then oxidized DCF was evaluated by flow cytometry. Representative histogram (**A**) and percentage of oxidized DCF positive cells (**B**) were shown (n = 3 for each group, *t* test). **C**, **D** relative MDA (**C**) and GSH (**D**) levels were evaluated in primary microglia treated as indicated for 24 h post OGD/R (n = 3 for each group, *t* test). **E**, **F** mice were treated with 5 mg/kg PPI, 10 mg/kg MHY1485, 10 mg/kg 3-MA or equal volume of vehicle control daily as indicated for one week after MCAO surgery, then microglia were separated from ischemic brain and stained with oxidized DCF for flow cytometry (n = 3 for each group, *t* test). Representative histogram (**E**) and percentage of oxidized DCF positive cells (**F**) were shown. **G**, **H** relative MDA (**G**) and GSH (**H**) levels were evaluated in microglia separated from ischemic brain of MCAO mice (n = 3 for each group, *t* test). **I**, **J** the protein expression of indicated genes in microglia treated as indicated was evaluated by western blot (**I**). Relative protein expression (**J**) was shown (n = 3 for each group, *t* test). *P < 0.05

Reactive oxidative species (ROS) are main intracellular stimuli for sustaining autophagy, and in turn, autophagy may promote ROS clearance by engulfing and degrading oxidized substance (Filomeni et al. 2015; Li et al. 2015). In the present study, ROS were measured by a fluorescent probe DCF-DA. PPI treatment significantly reduced the percentage of oxidized DCF positive cells in microglia after OGD/R, indicating that the level of ROS was decreased by PPI (Fig. 7A and B). Moreover, MHY1485 or 3-MA treatment largely abolished the influence of PPI on ROS levels, suggesting that inhibition of autophagy impaired PPI-mediated ROS clearance (Fig. 7A and B). In addition, PPI treatment reduced the level of MDA and increased the level of GSH in microglia after OGD/R, indicating that PPI suppressed lipid peroxidation and alleviated oxidative stress induced damage (Fig. 7C and D). However, these effects were also abrogated by MHY1485 or 3-MA treatment (Fig. 7C and D). The influence of PPI on autophagy-mediated ROS clearance was also evaluated in MCAO mice. PPI treatment evidently reduced the percentage of oxidized DCF positive cells in microglia separated from the ischemic brain of MCAO mice, but this was reversed by co-treating with MHY1485 or 3-MA (Fig. 7E and F). Similarly, the level of MDA was decreased while GSH increased in microglia separated from ischemic brain of MCAO mice treated with PPI, but this effect was abolished in microglia separated from ischemic brain of MCAO mice treated with PPI + MHY1485 or PPI + 3-MA (Fig. 7G and H). To further elucidate the underlying molecular mechanism of PPI-induced microglial polarization in MCAO mice, we focused on NLRP3 inflammasome. ROS is a major intracellular activator for NLRP3 inflammasome, while inflammasome formation plays important role in microglial polarization (Wu and Lu 2019). In the present study, the protein expression of NLRP3, caspase-1 and IL-1β was evaluated. NLRP3 is the core protein of NLRP3 inflammasome. Activation of NLRP3 inflammasome promoted caspase-1 activation and secretion of proinflammatory cytokine IL-1β in response to cellular damage or microbial infection (Kelley et al. 2019). We found that PPI treatment reduced NLRP3 expression and suppressed caspase-1 cleavage and IL-1β production in microglia after OGD/R or separated from ischemic brain of MCAO mice, indicating that PPI inhibited NLRP3 inflammasome activation (Fig. 7I and J). Moreover, co-treating with MHY1485 or 3-MA significantly attenuated the influence of PPI on NLRP3 inflammasome activation, with increased NLRP3 expression, caspase-1 cleavage and IL-1β production (Fig. 7I and J). Collectively, these results indicated that PPI facilitated autophagy-mediated ROS clearance to inhibit NLRP3 inflammasome activation in microglia.

### NLRP3 inflammasome reactivation by nigericin abolishes the effect of PPI on M2 microglia polarization

**Fig. 8:**
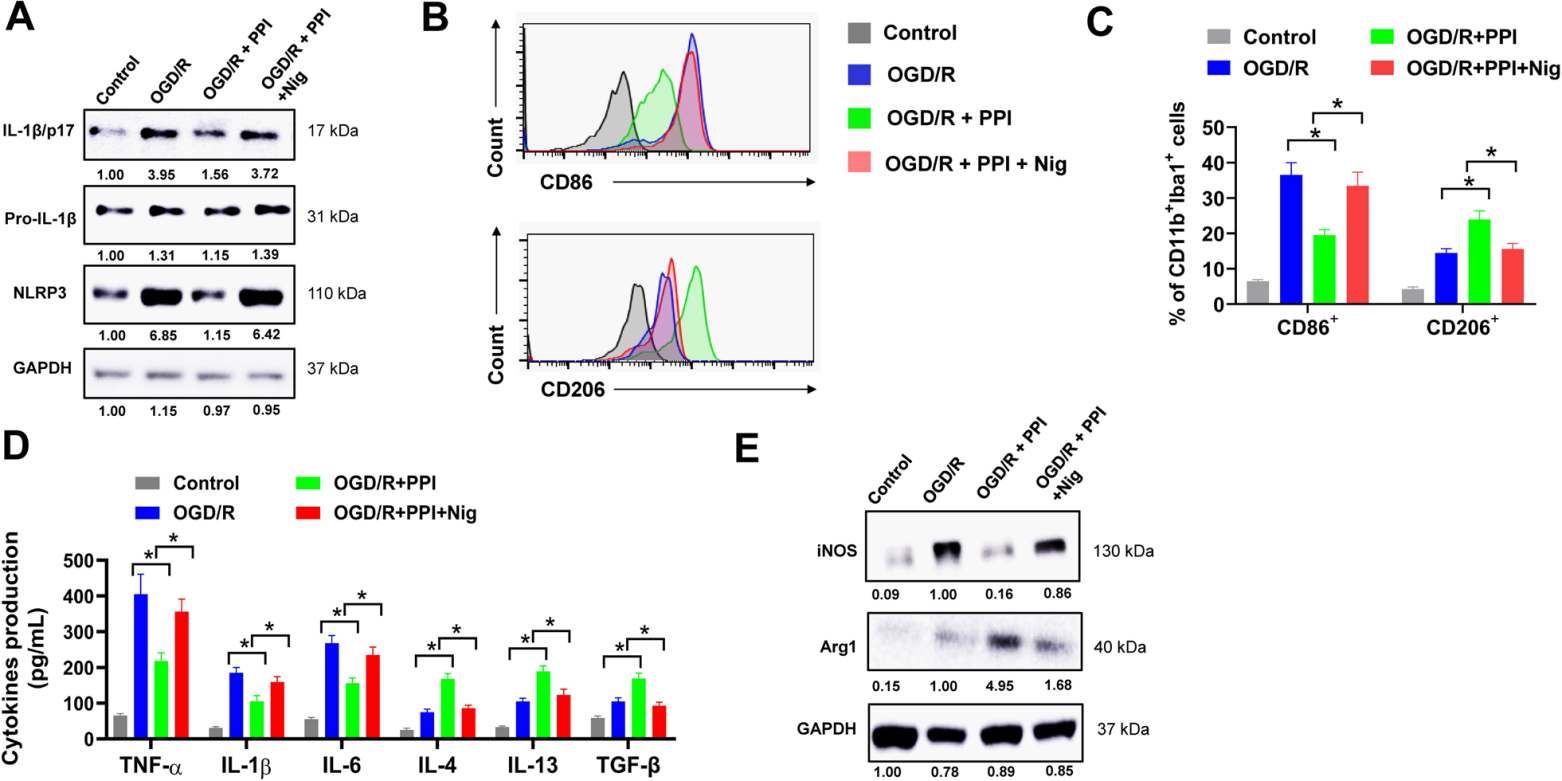
NLRP3 inflammasome reactivation by Nigericin abolishes the effect of PPI on M2 microglia polarization. **A**–**C** primary microglia isolated from healthy mice brain were treated with 2 μM PPI, 10 μM Nigericin (Nig) or equal volume of DMSO as indicated for 24 h post OGD/R, then collected lysates for western blot (**A**) or evaluated by flow cytometry (**B**, **C**). Representative histogram (**B**) and percentage of CD86^+^ and CD206^+^ cells in CD11b^+^Iba1^+^ microglia (**C**) were shown (n = 3 for each group, *t* test). **D** the secretion of pro-inflammatory cytokines (TNF-α, IL-1β, IL-6) and anti-inflammatory cytokines (IL-4, IL-13 and TGF-β) in culture medium of microglia after OGD/R was evaluated by ELISA assay (n = 3 for each group, *t* test). **E** the protein expression of iNOS and Arg1 in microglia post OGD/R was evaluated by western blot (n = 3 for each group, *t* test). *P < 0.05

To determine if PPI regulated microglial polarization via inhibiting NLRP3 inflammasome activation, we used the NLRP3 inflammasome activator nigericin. Co-treatment with nigericin successfully reversed the inhibitory effect of PPI on NLRP3 activation in microglia after OGD/R, with increased NLRP3 expression, caspase-1 cleavage and IL-1β production (Fig. 8A). Next, the influence of nigericin on microglial polarization was evaluated. PPI treatment apparently increased the percentage of CD11b^+^Iba1^+^CD206^+^ M2 microglia and decreased the percentage of CD11b^+^Iba1^+^CD86^+^ M1 microglia after OGD/R, but this was abrogated when co-treated with nigericin (Fig. 8B and C). In ELISA assay, the secretion of pro-inflammatory cytokines (TNF-α, IL-1β, IL-6) was evidently decreased while anti-inflammatory cytokines (IL-4, IL-13 and TGF-β) increased in culture medium of microglia by PPI treatment after OGD/R, but these effects were abolished when co-treated with nigericin (Fig. 8D). PPI treatment suppressed iNOS expression and increased Arg-1 expression, and this was reversed by nigericin (Fig. 8E). Taken together, these data indicated that NLRP3 inflammasome reactivation by nigericin abolishes the effect of PPI on M2 microglia polarization.

## Discussion

PPI, a natural ingredient extracted from *Paris polyphylla*, exhibits multiple bioactivities in a variety of diseases. PPI shows anti-cancer property in osteosarcoma by inhibiting growth and inducing apoptosis through inactivating Wnt/β-catenin pathway in vitro and in *vivo* (Chang et al. 2017). In 3XTg Alzheimer’s disease mice model, PPI reduces cognitive impairments and Alzheimer’s disease like pathology via suppressing CIP2A expression and PP2A re-activation (Zhou et al. 2020). In rheumatoid arthritis, PPI ameliorates synovial inflammation in the ankle joint by suppressing M1 macrophage polarization and T cell infiltration through NF-κB inhibition (Wang et al. 2018). In Propionibacterium acnes-induced inflammation, PPI suppresses secretion of proinflammatory cytokines IL-6, IL-8 and TNF-α, and attenuates the activation of NF-κB in keratinocytes (Zhu et al. 2019b). At present, there is no study reporting the potential influence of PPI on cerebral ischemia. Thus, we were aimed to determine if PPI treatment had any benefit on cerebral ischemia–reperfusion injury. In our study, PPI treatment alleviated cerebral ischemia–reperfusion injury and neuroinflammation, and improved functional recovery of mice after MCAO surgery, suggesting that PPI has the potential to develop as a novel drug for ischemic stroke treatment.

To further elucidate the underlying mechanism of PPI in cerebral ischemia, we found that PPI treatment modulated microglial polarization towards anti-inflammatory M2 phenotype. As the tissue-resident macrophage in brain, M2 microglia polarization plays an important role in controlling neuroinflammatory response and promoting tissue repair in cerebral ischemia. First, M2 microglia can secrete protective factors to prompt neuronal network repair via tissue and vascular remodeling. Next, M2 microglia may migrate to the injured hemisphere and mitigate the extent of neuroinflammation-induced injuries (Kanazawa et al. 2017). There are increasing evidences proving that certain drugs can regulate microglial polarization, thus show protective effects on cerebral ischemia–reperfusion injury. For example, loureirin B, a monomer compound extracted from Sanguis Draconis flavones (SDF), reduces infarct volume and neuron loss, and improves neuronal function recovery in MCAO rats by promoting M1 to M2 microglial polarization through STAT6/NF-κB signaling (Li et al. 2023c). Icaritin, a phytoestrogen extracted from the dried stems and leaves of *Epimedii*, suppresses inflammatory responses and protects against cerebral ischemia–reperfusion injury via facilitating M2 microglial polarization and inhibiting M1 microglial polarization in a rat cerebral ischemia model (Yu et al. 2022). Quercetin, a natural flavonoid exists in a variety of plants, ameliorates cerebral ischemia–reperfusion injury in a rat model of cerebral ischemia via inducing M2 microglial polarization (Li et al. 2023d). In our study, PPI treatment promoted M1 to M2 microglial phenotype shift in MCAO mice in vivo or after OGD/R in vitro, thus diminished neuroinflammation and promoted neuronal function recovery.

In the present study, PPI treatment promoted autophagy via suppressing Akt/mTOR signaling in microglia. Indeed, a number of studies suggest that PPI treatment may induce autophagy through Akt/mTOR inhibition. For example, PPI treatment induces autophagic cell death via inhibiting AKT/mTOR signaling, thus suppresses viability of colon cancer cells (Luo et al. 2022). In endometriosis, PPI inhibits growth and migration of endometrial stroma cells by inducing autophagy through Akt/mTOR signaling (Li et al. 2023b). In gastric carcinoma, PPI treatment facilitates autophagy and induces cell cycle arrest via restraining PDK1/Akt/mTOR signaling pathway in vitro and in vivo (He et al. 2019). Previous studies indicated that GSK-3α and GSK-3β are downstream targets of Akt and regulate mTOR and autophagy (Ryu et al. 2021; Zhou et al. 2013). Nevertheless, our data indicated that PPI had no apparent influence on total and phosphorylation of GSK-3α and GSK-3β, suggested that PPI might affect mTOR activity and autophagy directly through Akt signaling but not through the mediator GSK-3α and GSK-3β. The mTOR kinase is a major regulator of autophagic process, thus it is not surprised that reactivation of mTOR signaling by MHY1485 abolishes the inhibitory effect of PPI on autophagy. Furthermore, we found that inhibition of autophagy abrogated the effect of PPI on M2 microglial polarization. Actually, there are growing evidences demonstrating that autophagy is involved in microglial polarization after cerebral ischemia (Wang et al. 2023a). In eukaryotic cells, autophagy acts an important role in cell reprogramming by degrading large amount protein. During microglial polarization, transition from one phenotype to another phenotype is closely linked to quick degradation of protein associated with previous homeostatic state, thus it is reasonable that autophagy is involved in microglial polarization (Zubova et al. 2022). Indeed, lipopolysaccharide (LPS) can induce M1 microglial polarization and promote Akt/mTOR activation to suppress autophagic flow and enhance inflammatory response (Ye et al. 2020). In contrast, increased autophagy can inhibit LPS-induced M1 microglial polarization and inflammatory response by decreasing pro-inflammatory factors such as iNOS and IL-6 (Han et al. 2013). Furthermore, M2 microglial polarization is tightly connected with autophagy. For example, IL-4 treatment facilitates autophagy-dependent M2 microglial polarization and inhibits amyloid-β-induced M1 microglial polarization in Alzheimer's disease (Tang et al. 2019). Besides, blockage of autophagic flux suppresses the expression of M2-specific genes including Arg-1, IL-10 and Fizz1 (Yang et al. 2014). As PPI treatment increased autophagy in microglia, we speculated this might explain why PPI skewed M2 microglial polarization.

During cerebral ischemia, autophagy can be triggered by oxidative stress and inflammation (Zang et al. 2020). In return, properly controlled autophagy may promote ROS clearance and alleviate oxidative stress and inflammatory responses (Guo et al. 2020). For example, in cisplatin-induced spiral ganglion neuron damage, PRDX1 promotes autophagy through PTEN-Akt signaling, thus suppresses ROS accumulation and relieves cisplatin-induced oxidative stress (Liu et al. 2021b). In our study, the level of ROS was decreased by PPI treatment, but inhibition of autophagy by MHY1485 or 3-MA treatment largely abolished this effect, suggesting that PPI treatment facilitated autophagy-dependent ROS clearance. Moreover, we found that PPI treatment inhibited NLRP3 inflammasome activation in microglia, while NLRP3 inflammasome reactivation by nigericin abolished the effect of PPI on M2 microglia polarization. Actually, a number of studies demonstrate that NLRP3 inflammasome activation is involved in microglial polarization (Orihuela et al. 2016). In subarachnoid hemorrhage injury, SIRT1 facilitates M2 microglial polarization by suppressing ROS-mediated NLRP3 inflammasome activation, thus alleviates early brain injury (Xia et al. 2021). Argon is an inert gas that shows a favorable neuroprotective effect in a bulk of in vivo and in vitro models. During cerebral ischemia–reperfusion injury, argon application alleviates neuroinflammation via suppressing M1 microglial polarization and NF-κB/NLRP3 inflammasome signaling (Xue et al. 2023). In Alzheimer’s disease, TREML2 knockdown suppresses neuroinflammation by inhibiting NLRP3 inflammasome activation and inducing M2 microglial polarization in primary microglia (Wang et al. 2023b). Besides, we found that inhibition of autophagy by MHY1485 or 3-MA reversed the inhibitory effect of PPI on NLRP3 inflammasome activation. Indeed, in many diseases, inflammation and autophagy are intertwined and mutually influence each other (Plaza-Zabala et al. 2017). For example, disruption of autophagy enhances NLRP3 inflammasome activation in macrophage, accompanied by elevating ROS producing in mitochondria (Saitoh et al. 2008). In acute cerebral ischemia, autophagy negatively regulates the inflammatory response by acting a role in the inhibition of NLRP3-mediacted neuroinflammation (Fu et al. 2020). In traumatic brain injury, inhibition of autophagy in microglia leads to excessive neuroinflammation and failure in the degradation of NLRP3 inflammasome components (Hegdekar et al. 2023).

In summary, we found that PPI reduced infarct volume and neuroinflammation, and improved functional recovery of mice after MCAO. Moreover, PPI modulated microglial polarization towards anti-inflammatory M2 phenotype in MCAO mice or after OGD/R treatment. PPI promoted autophagy via suppressing Akt/mTOR signaling in microglia, while inhibition of autophagy abrogated the effect of PPI on M2 microglial polarization. Finally, PPI facilitated autophagy-mediated ROS clearance to inhibit NLRP3 inflammasome activation in microglia, and NLRP3 inflammasome reactivation by nigericin abolished the effect of PPI on M2 microglia polarization. Overall, our data elucidated a previously undiscovered function of PPI in cerebral ischemia–reperfusion injury. PPI might be a potential therapeutic drug for stroke.

### Supplementary Information

Supplementary Material 1. Figure S1. Pre-stroke PPI treatment alleviates cerebral ischemia–reperfusion injury and neuroinflammation in mice after MCAO. A, C57BL/6 mice were treated with indicated doses of PPI daily for 4 weeks, and body weight was monitored every week (n = 6 for each group, *t* test). B-G, MCAO mice were pre-treated with PPI or equal volume of vehicle control (Veh group) 24 h before MCAO surgery and lasted for 7 d, then histopathological changes of brain tissues were checked by H&E staining. Representative images (B) and denatured cell index (C) were shown. Apoptotic cells in mice brain were evaluated by TUNEL staining. Representative images (D) and relative TUNEL positive cells (E) were shown. Relative mRNA expression and secretion of indicated cytokines in ischemic brain tissues were evaluated by RT-qPCR (F) and ELISA assay (G) (n = 5 for each group, *t* test). *P < 0.05.

Supplementary Material 2. Figure S2. PPI shows no apparent influence on total and phosphorylation of GSK-3α and GSK-3β. A-B, primary microglia isolated from healthy mice brain were treated with PPI or equal volume of DMSO for 24 h post OGD/R, then collected lysates for western blot (A). Relative protein expression of indicated genes was shown (B). Cells in control group did not undergo OGD/R treatment but received equal volume of DMSO (n = 3 for each group, *t* test). C-D, MCAO mice were treated with PPI or equal volume of vehicle reagents (Veh group) daily for 7 d immediately after surgery, then microglia were separated from ischemic brain and collected lysates for western blot (C). Relative protein expression of indicated genes was shown (D) (n = 3 for each group, *t* test). *P < 0.05.

## Data Availability

The data that support the findings of this study are available on request from the corresponding author.
