# Supplementary figures and images for: Polyphyllin I alleviates neuroinflammation after cerebral ischemia–reperfusion injury via facilitating autophagy-mediated M2 microglial polarization
Source: Mol Med. 2024 May 14;30:59. doi: 10.1186/s10020-024-00828-5 (PMC11094947; doi:10.1186/s10020-024-00828-5)

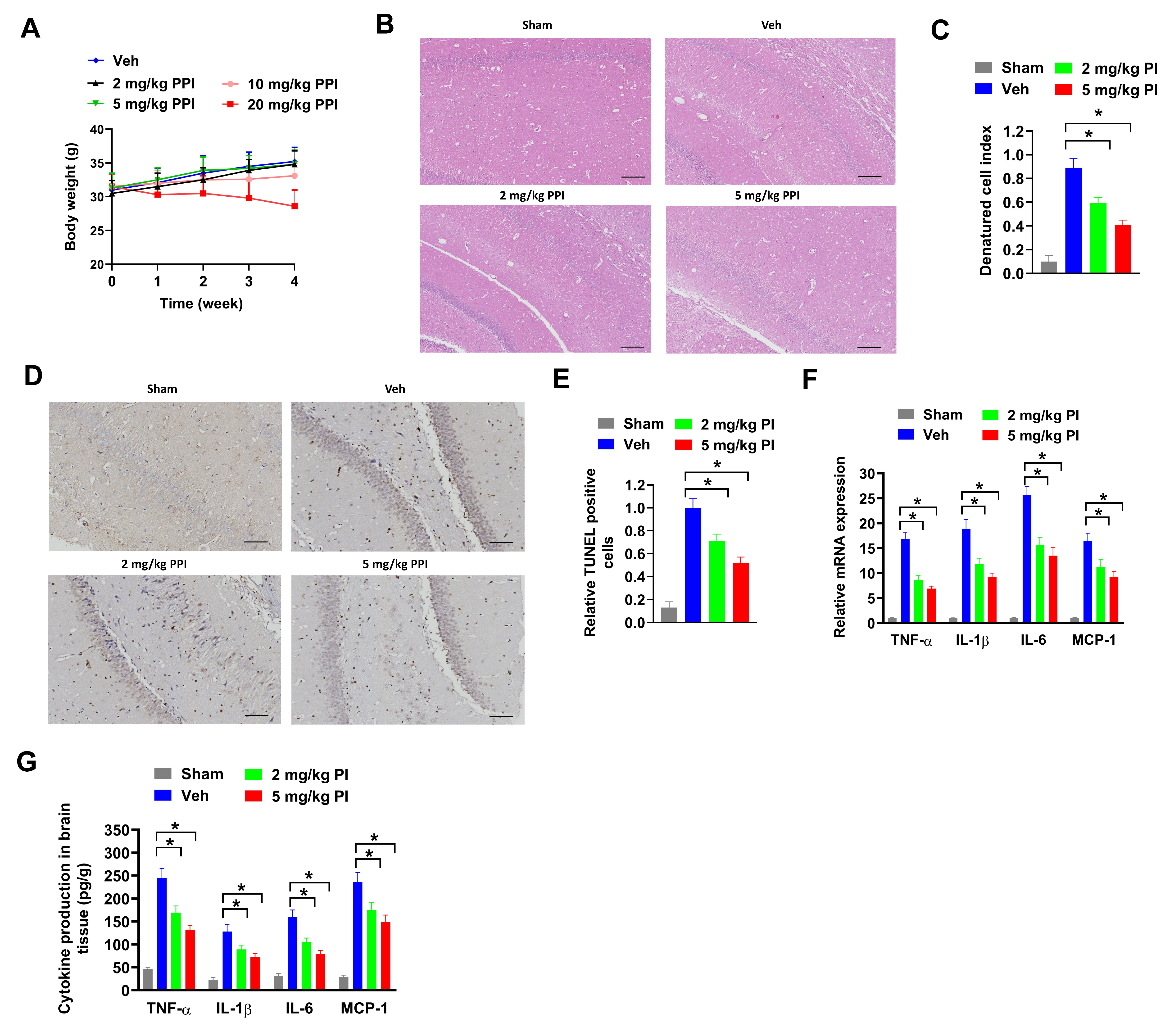

Supplement: Supplementary file 1 — Supplementary Material 1. Figure S1. Pre-stroke PPI treatment alleviates cerebral ischemia–reperfusion injury and neuroinflammation in mice after MCAO. A, C57BL/6 mice were treated with indicated doses of PPI daily for 4 weeks, and body weight was monitored every week (n = 6 for each group, t test). B-G, MCAO mice were pre-treated with PPI or equal volume of vehicle control (Veh group) 24 h before MCAO surgery and lasted for 7 d, then histopathological changes of brain tissues were checked by H&E staining. Representative images (B) and denatured cell index (C) were shown. Apoptotic cells in mice brain were evaluated by TUNEL staining. Representative images (D) and relative TUNEL positive cells (E) were shown. Relative mRNA expression and secretion of indicated cytokines in ischemic brain tissues were evaluated by RT-qPCR (F) and ELISA assay (G) (n = 5 for each group, t test). *P < 0.05. [file 10020_2024_828_MOESM1_ESM.tif]

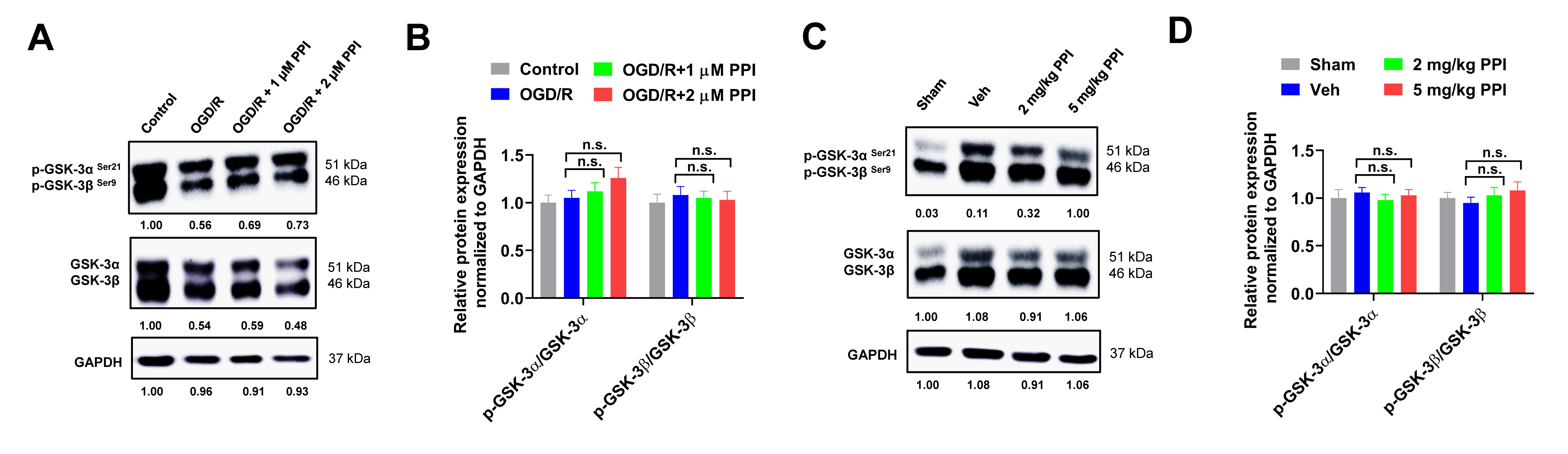

Supplement: Supplementary file 2 — Supplementary Material 2. Figure S2. PPI shows no apparent influence on total and phosphorylation of GSK-3α and GSK-3β. A-B, primary microglia isolated from healthy mice brain were treated with PPI or equal volume of DMSO for 24 h post OGD/R, then collected lysates for western blot (A). Relative protein expression of indicated genes was shown (B). Cells in control group did not undergo OGD/R treatment but received equal volume of DMSO (n = 3 for each group, t test). C-D, MCAO mice were treated with PPI or equal volume of vehicle reagents (Veh group) daily for 7 d immediately after surgery, then microglia were separated from ischemic brain and collected lysates for western blot (C). Relative protein expression of indicated genes was shown (D) (n = 3 for each group, t test). *P < 0.05. [file 10020_2024_828_MOESM2_ESM.tif]
